# Low-dose of organic trace minerals reduced fecal mineral excretion without compromising performance of laying hens

**DOI:** 10.5713/ajas.19.0270

**Published:** 2019-07-02

**Authors:** Jialing Qiu, Xintao Lu, Lianxiang Ma, Chuanchuan Hou, Junna He, Bing Liu, Dongyou Yu, Gang Lin, Jiming Xu

**Affiliations:** 1 Key Laboratory of Animal Nutrition and Feed in East China of Ministry of Agriculture and College of Animal Sciences, Zhejiang University, Hangzhou 310058, China; 2 Institute of Quality Standards and Testing Technology for Agricultural Products, Chinese Academy of Agricultural Sciences, Beijing 10081, China; 3 College of Life Sciences, Zhejiang University, Hangzhou 310058, China

**Keywords:** Fecal Mineral Excretion, Laying Hens, Organic Trace Minerals, Performance, Serum Indices, Tissue Mineral Retention

## Abstract

**Objective:**

The objective of this study was to investigate the effects of low doses of organic trace minerals (iron, copper, manganese, and zinc) on productive performance, egg quality, yolk and tissue mineral retention, and fecal mineral excretion of laying hens during the late laying period.

**Methods:**

A total of 405 healthy hens (HY-Line White, 50-week-old) were randomly divided into 3 treatments, with 9 replicates per treatment and 15 birds per replicate. The dietary treatments included feeding a basal diet + inorganic trace minerals at commercial levels (CON), a basal diet + inorganic trace minerals at 1/3 commercial levels (ITM), and a basal diet + proteinated trace minerals at 1/3 commercial levels (TRT). The trial lasted for 56 days.

**Results:**

Compared to CON, ITM decreased (p<0.05) egg production, daily egg mass, albumen height, eggshell strength, yolk Fe concentration, serum alkaline phosphatase activity and total protein, and increased (p<0.05) egg loss and feed to egg ratio. Whereas with productive performance, egg quality, yolk mineral retention, and serum indices there were no differences (p>0.05) between CON and TRT. The concentrations of Fe and Mn in the tissue and tibia were changed notably in ITM relative to CON and TRT. Both ITM and TRT reduced (p< 0.05) fecal mineral excretion compared to CON.

**Conclusion:**

These results indicate that dietary supplementation of low-dose organic trace minerals reduced fecal mineral excretion without negatively impacting hen performance and egg quality.

## INTRODUCTION

Trace minerals are essential to animals for growth and production [1]. Iron (Fe) has important physiological functions in animals, including participation in the composition and transport of hemoglobin carriers. As the activator of enzymes, iron is involved in the normal material metabolism and plays an important role in the process of intracellular oxidation [2]. And it is also the electron transporter of many important redox reactions, which involves physiological processes such as gene expression and cell proliferation [2]. As a component of the enzymes, copper (Cu), is directly involved in various metabolic processes. Maintaining the normal metabolism of iron and copper is conducive to the synthesis of hemoglobin and the maturation of red blood cells, meanwhile, copper is also involved in the physiological processes of reproduction, lactation, hair development, pigmentation, and bone development [3]. It is also an indispensable element for forming bone cells, collagen and elastin [3]. As an enzyme activator or component in the metabolism of carbohydrates, proteins, lipids and cholesterol, manganese (Mn) is an essential element for the growth of bone and reproduction within the animal [4]. In poultry, manganese is involved in the synthesis of eggshell acidic mucopolysaccharides and glycoproteins, which helps form the organic matrix of the eggshell, and is related to the degree of eggshell crack resistance. In addition, manganese participates in maintaining normal cell metabolism in antioxidant and immune systems [4,5]. Zinc (Zn) is a known necessary component and activating factor of more than 200 species of enzymes within animals, which increase the stability of the membrane structure, participate in a series of chemical reactions in the body, maintain normal metabolism and homeostasis [6]. Zinc can reduce animal oxidative stress and improve the body’s immunity, as well as supporting animal breeding, bone growth and development, it is also an important cofactor for carbonic anhydrase, playing an important role in the formation of eggshells [6,7]. Chen et al [8] reported that supplementation of 30 and 45 mg/kg of zinc in egg-laying ducks’ basic diets improved the egg production rate and feed utilization rate, and the antioxidant capacity was higher relatively. Singh et al [9] reported that failure to provide adequate Zn and Mn in the diet led to typical leg abnormalities in broilers and reduced serum alkaline phosphatase (AKP) activity.

Most mineral additives currently used in livestock produc tion come from inorganic compounds such as oxides, sulfates, carbonates and phosphates [5]. Antagonisms of dietary inorganic minerals can result in decreased absorption, leading to inorganic mineral salts being over supplied in commercial feeds to prevent deficiencies, which causes concern for the genetic potential of modern breeds and environmental pollution [10,11]. Organic trace minerals, which might promote emission reduction, have been shown to be more bioavailable than inorganic mineral salts and could be used at a lower level in animal feed [12,13]. Organic trace minerals are stable and not ionized prior to absorption, after entering the digestive tract, they can avoid the precipitation or adsorption by precipitants in the intestinal cavity. What’s more, chelating minerals are transported and absorbed as amino acids, therefore, the main benefit of using organic minerals is attributed to greater absorption, because they use the same absorption pathways as the amino acids to which they are bound, decreasing the competition for inorganic trace minerals binding sites, and thus reducing excretion through the bile and feces [9,11–13].

To the authors’ knowledge, there are few studies on the application of organic trace minerals in laying hens at present, therefore, the objective of the current experiment was to evaluate the effects of organic trace mineral complex completely replacing inorganic trace minerals on production performance, egg quality, trace minerals’ distribution in yolk, tissue, tibia, and excretion of laying hens during the late laying period.

## MATERIALS AND METHODS

### Animal care

Experimentation conformed to ethical norms. The use of experimental animals, and the procedures for animal management, and the collection of animal blood and tissues in this research were carried out according to the Institutional Animal Care and the Chinese Guidelines for Animal Welfare and approved by Animal Research Ethics Board of Zhejiang University.

### Birds and management

A total of 405 healthy hens (HY-Line White, 50-week-old) with similar body weight and egg-laying rates were used in the 56-day feeding trial. There were 3 dietary treatments and each treatment had 9 replicates with 15 birds per replicate pen. The dietary treatments included feeding a basal diet + inorganic trace minerals at commercial levels (CON: Fe, Cu, Mn, and Zn at 36, 12, 90, and 90 mg/kg, respectively. Which were designed on the basis of average survey data from major feed enterprises in China.), a basal diet + inorganic trace minerals at 1/3 commercial levels (ITM: Fe, Cu, Mn, and Zn at 12, 4, 30, and 30 mg/kg, respectively) and a basal diet + proteinated trace minerals (Bioplex PP, Alltech Inc., Nicholasville, KY, USA) at 1/3 commercial levels (TRT). The ration of basal supplemented diet was formulated according to NRC [14] recommendation to meet daily nutrient requirement of the hens for maintenance at the later laying period and modified to Chinese standards (NY/T 33–2004) [15]. The commercial trace mineral supplement levels were designed based on the average survey data from the major feed companies in China. The percentage of composition and nutrient level of basal diet are presented in Table 1. The contents of Fe, Cu, Mn, and Zn in the experimental diets were analyzed using inductively coupled plasma mass spectrometry (ICP-MS, model ELAN DRC-e, PerkinElmer Scientific Inc., Billerica, MA, USA), and are listed in Table 2. Hens were raised in the Key Laboratory of Animal Nutrition and Feed in East China of Ministry of Agriculture. In the same chicken house, the experimental hens were raised in a three-tiered three-dimensional cage (3 birds per cage, and each pen measured 45 cm ×30 cm ×30 cm) with feed and water *ad libitum*. Birds were standardized by weight and egg production before starting the experiment. Birds were subjected to 14 d of adjustment to the experimental diets. Lighting, temperature and humidity were set according to the conventional standards of commercial egg farms.

### Performance and egg quality

During the experiment, eggs were collected at 16:00 p.m. every day. Per replication unit, egg weight and the number of eggs (Including eggs with soft shell, broken shell, and different shape, etc.) produced were recorded daily to calculate the total egg weight, egg loss and egg production. Feed consumption was recorded every week during the feeding trial to calculate the average daily feed intake (ADFI, g/bird/d). Feed to egg ratio and daily egg mass were calculated according to total feedstuff consumption, total egg weight and egg production at the end of the experiment. Daily egg mass was calculated by multiplying the egg production rate (%) by the average weight of eggs (g) and dividing by 100 [5].

Per replicate, two eggs were collected randomly at the end of 8th week respectively, to assess the following parameters: albumen height, Haugh unit, yolk color, shell strength, and eggshell thickness. Subsequently the egg yolk was stored at −20°C for measuring trace mineral concentrations.

### Metabolites and enzyme activities in serum

On 57th day following the start of treatments, two birds from each replicate were randomly selected to collect blood samples. Blood samples (approximately 5 mL) were collected from the wing vein of the bird into individual gel and clot activator tubes before slaughter. Serum was obtained by centrifuging the clotted blood at 2,500×g at 4°C for 20 min and the supernatant was collected and stored at −80°C for later determination of metabolites and enzyme activities. Serum samples were analysed photometrically for AKP, glucose (GLU), serum albumin (ALB), total protein (TP), total cholesterol (T-CHO), and triglyceride (TG) in an automated biochemistry analyser using commercial kits (Nanjing Jiancheng Bioengineering Institute, Nanjing, China).

### Sample mineral analysis

Per replicate, one bird was humanely euthanized by cervical dislocation and tissue samples of liver, heart and breast muscle were removed, rinsed with double-distilled water and stored at −80°C for future mineral analysis.

Fat was extracted from tibias using ether after immersing the tibia in a boiling water bath for 30 min to remove the traces of flesh and cartilages. After washing with double-distilled water, the tibia was dried at 105°C for 24 h [9], and subsequently milled for mineral analysis.

Feces were collected for 3 consecutive days at the end of the trial for each pen. The collection of each 24 h was kept in polythene sachets and stored at −20°C and pooled at the end of the collection period pending analysis of Fe, Cu, Mn, and Zn. Then all fecal samples were dried in a hot-air oven at 65°C for 48 h, and ground to pass a 1-mm sieve to achieve homogenous samples for mineral analysis [16].

For mineral analysis, ground samples were diluted up to 50 mL with double-distilled water after wet ashing with nitric acid and hydrogen peroxide in a microwave digester (model MARS-5, version 194A07, Matthews, NC, USA) [16] for determination of Cu, Fe, Mn, and Zn. The Fe, Cu, Mn, and Zn concentrations in yolk, liver, heart, breast muscle and tibia were estimated using ICP-MS (model ELAN DRC-e, PerkinElmer Scientific Inc., USA), while feces were estimated using inductively coupled plasma optical emission spectrometry (ICP-OES, model Optima 8000DV, PerkinElmer Scientific Inc., USA). Bovine liver powder (GBW (E) 080193, National Institute of Standards and Technology, Beijing, China) was used as a standard reference material for verifying trace mineral analysis [16].

### Statistical analyses

The data obtained on performance, egg quality, metabolites, and enzyme activities in serum, tissue mineral status, and fecal mineral excretion were analyzed using IBM-SPSS 20.0 software (SPSS. Inc., Chicago, IL, USA). Normally distributed data were analyzed by one-way analysis of variance, and non-normally distributed data were analyzed by Kruskal-Wallis test. Level of significance was defined as p<0.05. Data were expressed as the mean±standard deviation.

## RESULTS

### Performance and egg quality

Performance (Table 3) and egg quality (Table 4) analysis showed that there were no differences (p>0.05) in ADFI, Haugh unit, yolk color, and eggshell thickness among the three treatments. Egg production, daily egg mass and shell strength in CON and TRT were significantly higher (p<0.05) than those in ITM, whereas egg loss and feed to egg ratio were lower (p<0.05) than those in ITM. There were no differences (p>0.05) in egg production, daily egg mass, feed to egg ratio, albumen height, and shell strength between CON and TRT. While, TRT reduced egg loss compared to CON.

### Metabolites and enzyme activities in serum

Data presented in Table 5 indicates that GLU, ALB, T-CHO, and TG were not significantly different (p>0.05) among the three treatments. Compared to CON and TRT, ITM decreased alkaline phosphatase activity (p<0.05) and TP in serum, however, there were no significant differences (p>0.05) in all serum indices between CON and TRT.

### Egg-yolk and tissue mineral status

Data in Table 6 indicate that yolk Fe concentration in ITM was significantly lower (p<0.05) than that in CON and TRT, however, there was no significant difference between CON and TRT. There were no significant differences (p>0.05) of Cu, Mn, and Zn levels in yolk among the three treatments.

As shown in Table 7, the concentrations of Fe, Cu, Mn, and Zn decreased significantly (p<0.05) in the liver in ITM compared to CON. Hens fed ITM diets had lower (p<0.05) Mn levels in the heart, compared to CON. The concentrations of Fe, Cu, Mn, and Zn in the breast muscle had no significant differences (p>0.05) among the three treatments. Whereas all trace mineral concentrations in CON and TRT had no significant differences (p>0.05).

### Tibia mineral deposition and fecal mineral excretion

As shown in Table 8, hens fed ITM diets had reduced Mn and Zn deposition in tibia, compared to CON and TRT. Both ITM and TRT reduced (p<0.05) fecal mineral excretion compared to CON (Table 8). ITM decreased Fe, Cu, Mn, and Zn by 54.25%, 43.73%, 35.18%, and 32.31%, respectively, compared to CON, and TRT decreased Fe, Cu, Mn, and Zn excretion by 60.77%, 57.99%, 52.62%, and 44.14%, respectively.

## DISCUSSION

This study indicates that dietary Fe, Cu, Mn, and Zn supplementation levels and sources do not affect ADFI, egg Haugh unit, yolk color, or eggshell thickness, which is consistent with previous studies [17,18]. Tabatabaie et al [19] found that compared to a Zn un-supplemented group, supplemental organic zinc as Albino-Zn at 25 or 50 mg/kg decreased feed intake, and increased albumen height and Haugh unit, but ZnSO_4_ in the same levels had minimal impact. In the present study, organic trace minerals supplemented at 1/3 commercial levels (TRT) had the same results in performance and egg quality compared to inorganic forms at commercial levels (CON). However, inorganic trace mineral (ITM) supplemented at the same dose as TRT decreased egg production, daily egg mass, albumen height and eggshell strength, and increased egg loss and feed to egg ratio compared to CON, which may affect commercial economic benefits.

Trace minerals Fe, Cu, Mn, and Zn are involved in growth and biochemical reactions of animal organisms [1]. Therefore, biochemical indicators and activities of enzymes in the blood can reflect the utilization of trace minerals [20]. Alkaline phosphatase is a zinc-containing enzyme, which is activated by manganese. And it is involved in calcification [21], deficiencies of Mn or Zn may cause subnormal activity of alkaline phosphatase, thus might depress bone and eggshell formation. In this regard, alkaline phosphatase activity in serum may serve as a good tool to compare the biological activities of trace minerals with different supplementation levels and sources. In our study, alkaline phosphatase activity in group ITM decreased by 15.12% and 13.47% compared to CON and TRT, respectively, however, TRT had the analogue result as the CON. In an earlier experiment, feeding broilers a basal diet without adding additional trace minerals resulted in typical leg abnormalities and a lower alkaline phosphatase activity in serum, while low doses of organic Fe, Cu, Mn, and Zn in two forms (methionine chelate- or yeast proteinate- based) didn’t affect these indicators, compared to inorganic forms at commercial level, which was in line with our results [9]. The contents of GLU, ALB, TP, T-CHO, and TG in serum may evaluate animal health. In our study, the low doses of trace minerals, regardless of inorganic and organic sources, didn’t alter these parameters except TP, compared to CON. Although ITM reduced the TP content, TRT showed no significant difference compared to CON. This indicated that low doses of organic trace minerals did not generate a negative impact on the health of laying hens.

The content of trace minerals in animal blood, liver, tibia and other tissues is commonly used as the main index to evaluate the biological efficiency of trace minerals in food [22]. The egg yolk, liver, heart, breast muscle, and tibia were chosen to assay in current study. Fe is stored primarily in liver, spleen, and bone [23], and its absorption rate can be changed by adjusting its amount and form in the feed. Our study found that ITM reduced Fe deposition in egg yolk and liver compared with CON, whereas Fe concentration of all tissues tested were consistent between TRT and CON. But they were inconsistent with earlier literature, which presented that Fe and Mn concentration in the liver, kidneys, and pancreas of broiler breeders didn’t change, regardless of mineral sources and levels [24], which might be due to the differences in type of animal species, experimental phase, or trace mineral level. The supplementation of Mn in diets can decrease malondialdehyde content in leg muscle by increasing mitochondrial manganese superoxide dismutase activity [25], consequently Mn deficiency might result in leg abnormality [9]. Previous research reported that augmenting dietary Mn increased eggshell thickness of hens [17]. Therefore, the reduction of manganese deposition in hens may affect their health and productivity. Our study indicated that Mn deposition in liver, heart and tibia of ITM hens were obviously lower than that in both CON and TRT, while no significant difference was found between TRT and CON. Some studies in other species have proved that the response to Zn intake increased linearly and then presented an inflection point when Zn intake met the requirement, hence tissue Zn concentrations are commonly used for assessing Zn requirements [26,27]. Previous literature also reported that tibia Zn was a sensitive and a suitable index for estimating Zn requirement of brown-egg laying hens [28]. In the current study, both liver and tibia Zn concentrations in ITM were significantly lower than that in CON and TRT. Bone is the site of mineral deposition and the change of mineral element content in the tibia can reflect the metabolic status of minerals within the animal [29]. It was interesting that while the reduction of inorganic Mn and Zn supplementation in late laying hens might cause abnormal maintenance of bone metabolism, the same low level of organic trace minerals had no adverse effects. Our results about Fe, Mn, and Zn deposition indicates that the low dose of organic trace minerals may be more efficient than the same level of inorganic trace minerals, since the organic form is more conducive to the deposition of trace minerals in the body. There were no significant differences in the deposition of Fe, Cu, Mn, and Zn in pectoral muscle, and no significant differences in copper deposition in all tissues, in the current trial. This might be due to muscle being the tissue with less trace mineral deposition, and small changes would be difficult to detect.

Inorganic trace minerals are widely used in livestock pro duction due to their easy accessibility and low cost, but their bioavailability is low. Thereby, in commercial production, producers generally supplement feedstuffs with a large safety margin in order to obtain higher economic benefits, thus causing a greater burden on the environment. It might improve bioavailability when trace minerals are chelated to a ligand, because the likelihood of interaction with other elements or combining with gastrointestinal antagonistic molecules is reduced [30]. Studies on replacing inorganic trace minerals with organic forms partially or entirely in animal feed have shown that organic trace minerals have higher bioavailability and reduced excretion in feces. An earlier study indicated that supplemented inorganic trace mineral with segmental organic modality could enhance the absorption coefficient of these trace minerals [31]. Nollet et al [32] reported that lower levels of organic trace minerals can be added to broiler diet to replace inorganic forms without any negative effects on broiler performance, even increasing feed conversion rate. Further, replacement with organic trace minerals resulted in significantly reduced concentrations of minerals in manure. The results of the current study manifested that both ITM and TRT significantly decreased fecal mineral excretion compared to CON, and TRT was more effective than ITM in reducing Cu, Mn, and Zn excretion, which are consistent with previous researches mentioned above. Since organic trace minerals have stable structure, they are absorbed by the intestinal brush border more effectively.

It was concluded from the present investigation that sup plemental organic Fe, Cu, Mn, and Zn as mineral proteinates at 1/3 commercial levels was superior to the same level of inorganic minerals. As environmental protection issues are urgently addressed, low doses of trace mineral proteinates can be used in poultry feed, reducing the risk of environmental contamination from fecal minerals without affecting performance and metabolic status of animals. Interestingly, in this study, hens fed organic trace minerals remarkably increased eggshell strength but not increased the eggshell thickness as compared to those fed identical level of inorganics. More research is needed to further explore the effect of organic trace minerals on the microstructure of eggshell. Further research is also needed to investigate the relationship between organic trace minerals and bone development.

## Figures and Tables

**Table 1:** Ingredient and nutrient composition of the basal diet

Items	Content
Ingredients (%)	
Corn	57.00
Soybean meal	24.00
CaCO_3_	8.50
Wheat middling	5.00
Emulsified fat powder	1.50
CaHPO_4_	1.00
Sodium chloride	0.30
DL-methionine	0.12
Lysine-HCl	0.08
Premix1)	2.50
Total	100.00
Nutrient levels2)	
ME (Mcal/kg)	2.63
CP	16.45
Lys	0.89
Met	0.45
Cys+Met	0.73
Ca	3.66
P	0.56
NPP	0.35

ME, metabolic energy; CP, crude protein; NPP, non-phytic acid phosphorus.

1) The premix without trace minerals provided the following per kg of the diet: vit A, 12,500 IU; vit D_3_, 4,125 IU; vit E, 15 IU; vit K 2 mg; thiamine, 1 mg; riboflavin, 8.5 mg; calcium pantothenate, 50 mg; niacin acid, 32.5 mg; pyridoxine, 8 mg; folic acid, 5 mg; vit B_12_, 5 mg; choline chloride, 500 mg; Se, 0.25 mg; I, 3 mg.

2) The analyzed Fe, Cu, Mn, and Zn contents in basal diet was 71.9, 7.2, 32.7, and 26.8 mg/kg, respectively. Except for crude protein, calcium and phosphorus levels, all other values were calculated.

**Table 2:** Assayed iron, copper, manganese, and zinc content in the experimental diets (mg/kg)

Ingredients	Treatment1)		

	CON	ITM2)	TRT3)
Iron	109.2±7.8 (36)4)	82.8±6.6 (12)	80.6±3.3 (12)
Copper	18.6±2.0 (12)	11.2±1.4 (4)	11.1±1.4 (4)
Manganese	123.3±5.5 (90)	64.7±5.9 (30)	66.4±3.4 (30)
Zinc	113.2±5.4 (90)	58.7±2.9 (30)	57.8±3.5 (30)

1) CON (control), a basal diet + inorganic trace minerals in commercial level; ITM, a basal diet + inorganic trace minerals in 1/3 commercial level; TRT, a basal diet + proteinated trace minerals in 1/3 commercial level.

2) Inorganic trace minerals provided by Zhejiang Weimeng Feed Technology Co., Ltd., Jiaxing, Zhejiang, China.

3) Organic source, proteinated trace minerals (Bioplex Poultry Pack, Alltech Inc., Nicholasville, KY, USA).

4) The values in brackets indicate the supplementation of trace minerals added to each group.

**Table 3:** Effect of experimental diets on productive performance of laying hens

Parameters	Treatments1)			p-value

	CON	ITM	TRT	
Egg production (%)	78.91±0.26^b^	76.08±1.40^a^	78.68±0.75^b^	<0.05
Daily egg mass (g/hen/d)	47.10±0.87^b^	45.73±0.81^a^	47.24±1.14^b^	<0.05
Egg loss (%)	2.07±0.10^b^	2.51±0.13^c^	1.91±0.16^a^	<0.05
ADFI (g/hen/d)	106.52±1.27	107.81±1.85	106.41±1.99	0.181
Feed to egg ratio	2.26±0.02^a^	2.36±0.04^b^	2.25±0.01^a^	<0.05

ADFI, average daily feed intake.

1) CON (control), a basal diet + inorganic trace minerals in commercial level; ITM, a basal diet + inorganic trace minerals in 1/3 commercial level; TRT, a basal diet + proteinated trace minerals in 1/3 commercial level.

a–c On the same line, values with no letter or the same superscripts mean no significant difference (p>0.05), whereas values with different superscript letters are significantly different (p<0.05).

**Table 4:** Effect of experimental diets on egg quality of laying hens

Parameters	Treatments1)			p-value

	CON	ITM	TRT	
Albumen height (mm)	8.34±0.69^b^	7.52±1.13^a^	8.02±0.79^ab^	<0.05
Haugh unit	83.90±5.14	81.30±6.19	81.42±7.30	0.380
Yolk color	6.00±0.84	5.67±0.49	5.83±0.71	0.361
Shell strength (N)	35.89±4.02^b^	24.87±3.59^a^	35.34±3.98^b^	<0.05
Eggshell thickness (mm)	0.35±0.01	0.34±0.03	0.35±0.03	0.532

1) CON (control), a basal diet + inorganic trace minerals in commercial level; ITM, a basal diet + inorganic trace minerals in 1/3 commercial level; TRT, a basal diet + proteinated trace minerals in 1/3 commercial level.

a,b On the same line, values with no letter or the same superscripts mean no significant difference (p>0.05), whereas values with different superscript letters are significantly different (p<0.05).

**Table 5:** Effect of experimental diets on serum indices of laying hens

Parameters	Treatments1)			p-value

	CON	ITM	TRT	
AKP (U/L)	348.48±13.02^b^	295.80±14.01^a^	341.84±25.72^b^	<0.05
GLU (mmol/L)	9.51±1.56	9.96±0.98	9.31±0.80	0.233
ALB (g/L)	21.76±1.11	22.55±2.01	21.98±1.70	0.342
TP (gprot/L)	35.83±3.26^b^	31.27±2.64^a^	34.88±1.90^b^	<0.05
T-CHO (mmol/L)	1.86±0.66	2.32±0.70	1.93±0.69	0.098
TG (mmol/L)	8.42±1.16	8.80±1.26	8.07±1.10	0.186

AKP, alkaline phosphatase; GLU, glucose; ALB, serum albumin; TP, total protein; T-CHO, total cholesterol; TG, triglyceride.

1) CON (control), a basal diet + inorganic trace minerals in commercial level; ITM, a basal diet + inorganic trace minerals in 1/3 commercial level; TRT, a basal diet + proteinated trace minerals in 1/3 commercial level.

a,b On the same line, values with no letter or the same superscripts mean no significant difference (p>0.05), whereas values with different superscript letters are significantly different (p<0.05).

**Table 6:** Effect of experimental diets on trace minerals concentration in egg-yolk of laying hens (mg/kg, as fresh basis)

Parameters	Treatments1)			p-value

	CON	ITM	TRT	
Fe	72.10±3.56^b^	58.07±9.09^a^	68.59±2.87^b^	<0.05
Cu	1.87±0.30	1.70±0.16	1.69±0.14	0.170
Mn	1.01±0.22	0.91±0.09	0.89±0.17	0.268
Zn	40.45±2.19	40.09±2.22	40.11±2.64	0.937

1) CON (control), a basal diet + inorganic trace minerals in commercial level; ITM, a basal diet + inorganic trace minerals in 1/3 commercial level; TRT, a basal diet + proteinated trace minerals in 1/3 commercial level.

a,b On the same line, values with no letter or the same superscripts mean no significant difference (p>0.05), whereas values with different superscript letters are significantly different (p<0.05).

**Table 7:** Effect of experimental diets on tissue (liver, heart, and breast muscle) trace mineral concentrations of laying hens (mg/kg, as fresh basis)

Parameters	Treatments1)			p-value

	CON	ITM	TRT	
Liver				
Fe	164.11±5.09^b^	119.56±11.24^a^	156.00±16.11^b^	<0.05
Cu	5.20±0.80	4.98±0.58	5.22±0.66	0.771
Mn	5.26±0.42^b^	3.68±0.37^a^	5.06±0.51^b^	<0.05
Zn	72.59±9.68^b^	59.96±5.86^a^	67.85±8.83^ab^	<0.05
Heart				
Fe	68.05±5.18	66.85±7.90	65.87±4.95	0.756
Cu	6.08±1.16	5.47±1.06	5.52±0.73	0.379
Mn	1.09±0.14^b^	0.87±0.03^a^	1.03±0.13^b^	<0.05
Zn	24.69±1.66	23.34±0.98	23.99±1.52	0.151
Breast muscle				
Fe	14.41±1.98	14.14±2.35	14.54±1.73	0.914
Cu	0.61±0.08	0.57±0.05	0.58±0.04	0.330
Mn	0.24±0.04	0.23±0.04	0.22±0.03	0.362
Zn	7.15±0.35	6.93±0.41	6.95±0.42	0.449

1) CON (control), a basal diet + inorganic trace minerals in commercial level; ITM, a basal diet + inorganic trace minerals in 1/3 commercial level; TRT, a basal diet + proteinated trace minerals in 1/3 commercial level.

a,b On the same line, values with no letter or the same superscripts mean no significant difference (p>0.05), whereas values with different superscript letters are significantly different (p<0.05).

**Table 8:** Effect of experimental diets on trace minerals concentration in the tibia and feces of laying hens (mg/kg, as air-dry basis)

Parameters	Treatments1)			p-value

	CON	ITM	TRT	
Tibia				
Fe	82.45±3.14	80.44±3.40	80.45±3.76	0.378
Cu	1.29±0.21	1.40±0.15	1.32±0.21	0.481
Mn	8.43±0.51^b^	6.86±0.30^a^	8.36±0.83^b^	<0.05
Zn	159.38±6.01^b^	136.97±5.21^a^	157.61±7.18^b^	<0.05
Feces				
Fe	1,591.24±81.59^b^	727.99±62.30^a^	624.18±30.09^a^	<0.05
Cu	91.25±1.65^c^	51.35±4.83^b^	38.33±5.86^a^	<0.05
Mn	751.86±7.33^c^	487.38±30.31^b^	356.25±9.28^a^	<0.05
Zn	615.83±8.61^c^	416.87±12.09^b^	343.98±26.52^a^	<0.05

1) CON (control), a basal diet + inorganic trace minerals in commercial level; ITM, a basal diet + inorganic trace minerals in 1/3 commercial level; TRT, a basal diet + proteinated trace minerals in 1/3 commercial level.

a–c On the same line, values with no letter or the same superscripts mean no significant difference (p>0.05), whereas values with different superscript letters are significantly different (p<0.05).

## References

[1] Richards JD, Zhao J, Harrell RJ, Atwell CA, Dibner JJ (2010). Trace mineral nutrition in poultry and swine. Asian-Australas J Anim Sci.

[2] Han O (2011). Molecular mechanism of intestinal Iron absorption. Metallomics.

[3] Kim YH, Yoo JS, Park JC (2008). Effects of Copper and Zinc sources on growth performance, nutrient digestibility, carcass traits and meat characteristics in finishing pigs. Korean J Food Sci An.

[4] Aschner JL, Aschner M (2005). Nutritional aspects of Manganese homeostasis. Mol Aspects Med.

[5] Stefanello C, Santos TC, Murakami AE, Martins EN, Carneiro TC (2014). Productive performance, eggshell quality, and eggshell ultrastructure of laying hens fed diets supplemented with organic trace minerals. Poult Sci.

[6] Park SY, Birkhold SG, Kubena LF, Nisbet DJ, Ricke SC (2004). Review on the role of dietary Zinc in poultry nutrition, immunity, and reproduction. Biol Trace Elem Res.

[7] Qian L, Yue X, Hu L, Ma Y, Han X (2016). Changes in diarrhea, nutrients apparent digestibility, digestive enzyme activities of weaned piglets in response to chitosan-zinc chelate. Anim Sci J.

[8] Chen W, Wang S, Zhang HX (2017). Optimization of dietary Zinc for egg production and antioxidant capacity in Chinese egg-laying ducks fed a diet based on corn-wheat bran and soybean meal. Poult Sci.

[9] Singh AK, Ghosh TK, Haldar S (2015). Effects of methionine chelate- or yeast proteinate-based supplement of copper, iron, manganese and zinc on broiler growth performance, their distribution in the tibia and excretion into the environment. Biol Trace Elem Res.

[10] Aksu T, Ozsoy B, Aksu DS, Yoruk MA, Gul M (2011). The effects of lower levels of organically complexed zinc, copper and manganese in broiler diets on performance, mineral concentration of tibia and mineral excretion. Kafkas Univ Vet Fak Derg.

[11] Leeson S, Caston L (2008). Using minimal supplements of trace minerals as a method of reducing trace mineral content of poultry manure. Anim Feed Sci Technol.

[12] Liu Y, Ma YL, Zhao JM, Vazquez-Anon M, Stein HH (2014). Digestibility and retention of zinc, copper, manganese, iron, calcium, and phosphorus in pigs fed diets containing inorganic or organic minerals. J Anim Sci.

[13] Mabe I, Rapp C, Bain MM, Nys Y (2003). Supplementation of a corn-soybean meal diet with manganese, copper, and zinc from organic or inorganic sources improves eggshell quality in aged laying hens. Poult Sci.

[14] National Research Council (1994). Nutrient requirements of poultry: 9th Revised Edition.

[15] The Ministry of Agriculture of the People’s Republic of China (2004). Feeding standard of chicken.

[16] Liu B, Xiong P, Chen N (2016). Effects of replacing of inorganic trace minerals by organically bound trace minerals on growth performance, tissue mineral status, and fecal mineral excretion in commercial grower-finisher pigs. Biol Trace Elem Res.

[17] Xiao JF, Wu SG, Zhang HJ (2015). Bioefficacy comparison of organic manganese with inorganic manganese for eggshell quality in Hy-Line Brown laying hens. Poult Sci.

[18] Zhang YN, Wang J, Zhang HJ, Wu SG, Qi GH (2017). Effect of dietary supplementation of organic or inorganic manganese on eggshell quality, ultrastructure, and components in laying hens. Poult Sci.

[19] Tabatabaie MM, Aliarabi H, Saki AA, Ahmadi A, Hosseini Siyar SA (2007). Effect of different sources and levels of zinc on egg quality and laying hen performance. Pak J Biol Sci.

[20] McLaren G, Patel K, Said H, Anderson G, Said H (2015). Differentiation-dependent regulation of intestinal iron absorption: cell and molecular mechanisms. FASEB J.

[21] Debernard B, Gherardini M, Lunazzi GC (1985). Biochemical and morphologic evidence that alkaline phosphatase of matrix vesicles is involved in calcification. Bone.

[22] Martin RE, Mahan DC, Hill GM, Link JE, Jolliff JS (2011). Effect of dietary organic microminerals on starter pig performance, tissue mineral concentrations, and liver and plasma enzyme activities. J Anim Sci.

[23] Taschetto D, Vieira SL, Angel CR (2017). Iron requirements of broiler breeder hens. Poult Sci.

[24] Wang G, Liu LJ, Wang ZP (2019). Comparison of inorganic and organically bound trace minerals on tissue mineral deposition and fecal excretion in broiler breeders. Biol Trace Elem Res.

[25] Lu L, Ji C, Luo XG, Liu B, Yu SX (2006). The effect of supplemental manganese in broiler diets on abdominal fat deposition and meat quality. Anim Feed Sci Technol.

[26] Hunt JR, Johnson LK (1992). Dietary protein, as egg albumen: effects on bone composition, zinc bioavailability and zinc requirements of rats, assessed by a modified broken-line model. J Nutr.

[27] Mohanna C, Nys Y (1999). Effect of dietary zinc content and sources on the growth, body zinc deposition and retention, zinc excretion and immune response in chickens. Br Poult Sci.

[28] Qin S, Lu L, Zhang X (2017). An optimal dietary zinc level of brown-egg laying hens fed a corn-soybean meal diet. Biol Trace Elem Res.

[29] Swiatkiewicz S, Arczewska-Wlosek A, Jozefiak D (2015). Bone quality, selected blood variables and mineral retention in laying hens fed with different dietary concentrations and sources of calcium. Livest Sci.

[30] Manangi MK, Vazquez-Anon M, Richards JD, Carter S, Buresh RE, Christensen KD (2012). Impact of feeding lower levels of chelated trace minerals versus industry levels of inorganic trace minerals on broiler performance, yield, footpad health, and litter mineral concentration. J Appl Poult Res.

[31] Mondal S, Haldar S, Saha P, Ghosh TK (2010). Metabolism and tissue distribution of trace elements in broiler chickens’ FED diets containing deficient and plethoric levels of copper, manganese, and zinc. Biol Trace Elem Res.

[32] Nollet L, Van der Klis JD, Lensing M, Spring P (2007). The effect of replacing inorganic with organic trace minerals in broiler diets on productive performance and mineral excretion. J Appl Poult Res.

